# High-intensity resistance training improves quality of life, muscle endurance and strength in patients with myositis: a randomised controlled trial

**DOI:** 10.1007/s00296-024-05698-y

**Published:** 2024-08-27

**Authors:** Kasper Yde Jensen, Per Aagaard, Charlotte Suetta, Jakob Lindberg Nielsen, Rune Dueholm Bech, Henrik Daa Schrøder, Jan Christensen, Casper Simonsen, Louise Pyndt Diederichsen

**Affiliations:** 1 Copenhagen Research Center for Autoimmune Connective Tissue Diseases (COPEACT), Center for Rheumatology and Spine Diseases, Rigshospitalet, Copenhagen, Denmark; 2 Department of Sports Science and Clinical Biomechanics, University of Southern Denmark, Odense, Denmark; 3 Geriatric Research Unit, Department of Geriatric and Palliative Medicine, Copenhagen University Hospital, Bispebjerg, Frederiksberg, Denmark; 4 Department of Orthopaedics and Traumatology, Zealand University Hospital, Koege, Denmark; 5 Department of Pathology, Odense University Hospital, Odense, Denmark; 6 Department of Occupational Therapy and Physiotherapy, Copenhagen University Hospital - Rigshospitalet, Copenhagen, Denmark; 7 Centre for Physical Activity Research, Copenhagen University Hospital - Rigshospitalet, Copenhagen, Denmark; 8 Department of Rheumatology, Odense University Hospital, Odense, Denmark; 9 Department of Clinical Medicine, Faculty of Health and Health Sciences, University of Copenhagen, Copenhagen, Denmark

**Keywords:** Autoimmune disease, Clinical trials, Intervention, Physical activity, Quality of life, Rheumatology

## Abstract

Myositis is associated with reduced quality of life, which is accompanied by significant impairments in muscle endurance and strength, altogether representing cardinal traits in patients with myositis. This randomised controlled trial aimed to investigate the effect of high-intensity resistance training on quality of life in patients with myositis. Thirty-two patients with established, stable myositis were randomised to 16 weeks of high-intensity resistance training (intervention group) or 16 weeks of usual care (control group). Primary outcome was quality of life assessed as the change in the physical component summary score (PCS) of the Short Form-36 health questionnaire from baseline to post-intervention. Secondary outcomes included functional capacity measures, such as functional index 3, and International Myositis Assessment and Clinical Studies Group (IMACS) disease activity and damage core set measures, including manual muscle testing 8 (MMT8). The primary outcome PCS showed an improvement in favour of high-intensity resistance training with a between-group difference of 5.33 (95% CI 0.61; 10.05) (p = 0.03). Additionally, functional index 3 showed a between-group difference indicating greater gains with high-intensity resistance training 11.49 (95% CI 3.37; 19.60) (p = 0.04), along with a between-group improvement in MMT8 1.30 (95% CI 0.09; 2.51) (p = 0.04). High-intensity resistance training for 16 weeks effectively improved quality of life in patients with myositis. Clinical measures of muscle endurance and muscle strength were also found to improve with high-intensity resistance training, while patients stayed in disease remission. Consequently, progressively adjusted high-intensity resistance training is feasible and causes no aggravation of the disease, while benefitting patients with myositis.

Clinical trial registration: Clinicaltrials.gov ID: NCT04486261—https://clinicaltrials.gov/study/NCT04486261.

## Introduction

Idiopathic inflammatory myopathies—known as myositis—comprise a heterogeneous group of rare, autoimmune muscle diseases that share cardinal features such as muscle inflammation, muscle weakness and decreased muscle endurance [1, 2]. Prednisolone and other immunosuppressive medications can decrease disease activity in patients with myositis, except in patients with sporadic inclusion body myositis (sIBM) [3]. Even though immunosuppressive treatment is effective, muscle weakness persists and functional capacity remains reduced compared to pre-disease levels [4]. With the decline in muscle strength and endurance, activities of daily living become more taxing, in turn negatively affecting quality of life (QoL) [5–9]. Indeed, despite immunosuppressive treatment, long-term follow-up in patients with myositis has demonstrated reduced QoL compared to healthy age-matched adults [8, 10, 11]. Therefore, non-pharmacological adjunct treatment paradigms should be explored in this patient population.

Physical exercise is generally considered beneficial for overall health [12], however, there are significant gaps in knowledge concerning the effects of exercise interventions in patients with myositis. Only four randomised controlled trials (RCT) with exercise-based interventions have been performed over the last 25 years (excl. sIBM) [13–16], and the effect of isolated (i.e. stand-alone) high-intensity resistance training has not previously been examined in patients with myositis in an RCT design. This lack of investigation is particularly striking since high-intensity resistance training (i.e., weights > 70% of 1 repetition maximum (RM), is recognized for mitigating the loss of muscle strength across various healthy and patient populations and age groups, in turn eliciting significant improvements in functional capacity. Previous non-RCT training studies in myositis patients have included resistive/resistance (i.e., low intensity) exercises as a part of multi-exercise component training protocols [14, 16–20]. These studies show promising results in terms of increased muscle strength, and signs of attenuated physical impairments, along with unaltered levels of inflammation within the trained muscles [14, 16–20]. However, due to the generally low intensity of the resistive training performed, as well as it being combined with endurance exercises, it is difficult to ascertain the specific effect(s) of the resistance exercises per se on functional capacity and QoL in patients with myositis. Furthermore, only two of these multi-exercise studies included assessments of QoL [16, 20].

Consequently, the primary aim of the present study was to investigate the efficacy of high-intensity resistance training on QoL in patients with myositis. As a secondary aim, we investigated the effect of high-intensity resistance training on functional capacity, maximal leg extensor muscle power, and various measures from the International Myositis Assessment and Clinical Studies Group (IMACS) Disease Activity Core Set Measures.

## Materials and methods

### Study design

The present study was conducted as a single-centre, single-blinded superiority RCT (Clinicaltrials.gov ID: NCT04486261) [21]. The study was conducted at the Copenhagen University Hospital—Rigshospitalet. The study employed a two-armed (1:1) study design with 16 weeks of high-intensity resistance training (HRT) in the intervention group (IG) or usual care in the control group (CG). The resistance training was conducted at the Department of Occupational Therapy and Physiotherapy at Rigshospitalet, with a small subgroup of patients (n = 4) trained at the Department of Physiotherapy at Zealand University Hospital, Køge. All training was supervised by the principal investigator (KYJ).

Separate pre-test sessions were performed prior to baseline measurements to familiarize the study participants with the functional and muscle-related tests. Outcome variables were obtained at baseline and following the 16-week intervention period. Three assessors (chief physician: LPD, physiotherapists: CG and KYJ) performed all data measurements at pre- and post-training, while blinded to group allocation, except KYJ who trained the patients and served as assessor muscle endurance—Functional index 3.

The study was approved by The Danish National Committee on Health Research Ethics (H-20030409) and The Danish Data Protection Agency (P-2020–553). Further, the study was conducted in accordance with the Declaration of Helsinki and SPIRIT guidelines [22]. All study participants provided their written informed consent before engaging in the study.

### Patient involvement

A patient advisory board was established to ensure that narrative patient inputs were included in the present project [23, 24]. This advisory board participated in discussions on ethics, study design, relevance, and feasibility. The advisory board was not involved in the data interpretation, writing, or editing of the present article.

### Participants and randomisation

Between February 2021 and May 2021, 160 patients aged ≥ 18 years fulfilling the criteria for idiopathic inflammatory myopathies by EULAR/ACR [25, 26] and affiliated to a tertiary care centre for myositis in the Capital Region of Denmark, were screened for eligibility by a consultant (chief physician LPD) experienced in patients with myositis. Patients were excluded if: affected by additional systemic autoimmune disease (except Sjögrens Syndrome), sIBM, had co-morbidity preventing resistance training (severe heart/lung-disease, uncontrolled hypertension (systolic > 160 mmHg, diastolic > 100 mmHg), severe knee/hip arthritis) [21], alcohol- and/or drug abuse.

Patients should have a diagnosis for ≥ 6 months and be in stable background immunosuppressive treatment for at least 1 month before entering the study, receiving ≤ 5 mg of oral prednisolone daily. Adjustments in dose or type of background immunosuppressives were not permitted during the time course of the study.

Stratified group allocations were performed following baseline testing and conducted by the investigator in charge of training (KYJ), to ensure all other assessors stayed blinded. Allocation procedures were performed using a randomised allocation program (Sealed Envelope Ltd. 2021) with a 1:1 block randomisation with stratifications [age (< 50 or ≥ 50 years of age) and lung involvement (yes or no)].

### Intervention procedures

#### High-intensity resistance training

In addition to the usual care, participants allocated to training received supervised HRT twice a week for 16 weeks, with a duration of ~ 1 h for each session.

Warm-up was performed for 10 min on a stationary bike at a low to moderate pace (70–80 rpm) and low-to-moderate intensity (50–100 W).

The training protocol consisted of five resistance exercises: horizontal bench press, horizontal leg press, seated rows, and knee extension. Seated biceps curls were performed with participants sitting on a chair.

To determine the individual training intensity at baseline, a 5 repetitions maximum (RM) test was performed prior to the start of training by all participants.

Sessions 1–4 focused on familiarisation and preconditioning to the designated training protocol, with exercises performed in three sets of 10 repetitions at a target intensity of 15RM. Intensity (i.e., external loads) was increased in the fifth session to match individual 10RM.

Exercise loads were progressively adjusted throughout the intervention period to ensure that loading intensity was maintained at 10RM. Training loads were increased when the participant completed two extra repetitions (i.e., 12 reps) in the last set (i.e., third) of the respective exercise. Inter-set pauses were 60–90 s. Training loads and volumes for all training sessions were recorded in a training diary for each participant (training data included in Results).

#### Usual care

The control group received usual care and attended their regular consultations with their respective physicians, further they were instructed to retain habitual physical activity levels throughout the intervention period. There were no study-related contacts (i.e., phone calls, emails, visits) during the intervention period.

### Outcome assessments

Patient demographics and disease characteristics were assessed at baseline. Demographical information was extracted from the electronic medical records. Primary and secondary outcome variables were measured at baseline and following the 16-week intervention period (3–7 days following the last training session for IG). Adverse events (AEs) were registered throughout the intervention period. Aes were divided into “non-serious” and “serious”, with subcategories: “expected”, “unexpected” as well as “study-related”, “possibly study-related” and “not study related”. Training-related AEs (in the intervention group) were noted systematically in the individual training diaries. Principal investigator (KYJ) and chief physician (LPD) together decided on the category and subcategories of each AE.

#### Quality of life

As recommended by IMACS [27], QoL was assessed by the Short Form-36 health questionnaire (SF-36), which has been found to have good construct and criterion validity in patients with myositis [28].

The primary outcome variable was change in physical component summary SF-36 score (PCS) from baseline to post-intervention. PCS consists of four subscales: physical functioning, bodily pain, general health, and role physical. The secondary outcome mental component summary score (MCS) of SF-36 consists of four subscales: vitality, social functioning, role emotional, and mental health. Each of the eight subscales scores from 0 to 100, with higher scores representing better health status [29].

#### Functional capacity, muscle power and strength

Muscle endurance was measured utilizing the functional index 3 (FI3), which is a validated tool for patients with myositis [30]. In brief, the test consists of three tasks (shoulder flexion, neck flexion, and hip flexion), all performed at a pace of 40 beats per minute controlled by a digital metronome. Completed repetitions were counted, until participants were unable to follow the external count due to fatigue or had completed a maximum of 60 (+ 5 initial “learning”) repetitions. Shoulder and hip flexions were performed for both the dominant and non-dominant sides, respectively, while always starting with the dominant side.

As a functional measure of lower extremity strength and short-term endurance, the 30-s Sit-to-Stand (30-s STS) test was implemented [31]. In brief, participants were seated in a rigid chair (45-cm vertical seat position) and asked to stand up and sit down as many times as possible in 30 s. The use of arms for assistance was not allowed and hips and knees had to be fully extended at the top of each rise.

Timed up & go (TUG) testing was performed to assess horizontal gait function and dynamic postural balance. TUG is considered a valid and reliable way of evaluating functional mobility [32]. Participants were instructed to rise from a chair (45-cm vertical seat position) with no arm assistance, walk as fast as possible 3-m forward, turn around a cone, walk back, and sit down. Three attempts were completed, and the fastest (shortest) time was selected for analysis.

As an additional measure of gait function, participants also performed the 2-min walk test (2MWT), which is validated as a reliable test of muscle endurance and is highly associated with changes in muscle strength [33]. The test was performed on a 20-m long indoor track and participants were instructed to cover the longest distance possible in 2 min.

Standing postural balance was tested by a static balance test, which is a part of the Short physical performance battery [34]. The test consists of three 10-s static stances, with feet-position gradually becoming more challenging (feet together, semi-tandem, and full tandem), with no assistance from arms or assistive devices. The test was stopped when the participants completed the three positions or if the participant had to adjust the position of the feet due to balance loss. The score range was 0–30 s, with a higher score (longer time) representing better balance [34].

Maximal unilateral leg extensor muscle power (LEP) was assessed for the dominant limb using the Nottingham power rig [35]. The participant was seated in the power rig with the arms crossed over the chest and the dominant leg at a position that allowed for a 15° angle in the knee when the footplate was fully extended [36]. At least five attempts were completed separated by 30-s rest periods, with additional attempts performed until no further increase in peak power output could be observed in two successive trials. The best attempt (highest peak power) was selected for statistical analysis.

In IG, a 10RM test was performed at the 5th and the 32nd training sessions (last session) for each of the five training exercises.

#### IMACS disease activity and disease damage core set measures

The disease activity core set measures by IMACS represent a validated, standardized tool to assess disease activity in patients with myositis and was implemented in the present study [27].

Physician global activity (PhGA), patient global activity (PtGA), and extramuscular global assessment (EMGA) were evaluated using a visual analogue scale (0–100-mm, VAS; with higher scores representing higher disease activity). Manual muscle testing 8 (MMT8) evaluated static muscle strength (against manual resistance applied by the tester) in eight predefined muscle groups (deltoid middle, biceps brachii, wrist extensors, quadriceps femoris, ankle dorsiflexors, neck flexors, gluteus medius, gluteus maximus) and was graded from 0 to 10, with a maximum score of 80 unilaterally (higher scores represent higher strength). Health assessment questionnaire (HAQ) assessed the participants perceived physical function (rating from 0 to 3, with lower scores representing better physical function). Plasma creatine kinase (CK) was measured by venous blood sampling (taken prior to physical testing at baseline and at least 72 h following last training session at post-intervention).

The IMACS disease damage core set measures consist of physician global damage (PhGD) and patient global damage (PtGD) and were evaluated using the VAS scale (0–100 mm; with higher scores representing more damage) [27].

### Statistical analysis

#### Sample size

The study was designed as a superiority trial. We hypothesized to observe a group mean change of ≥ 20% in our primary outcome variable (PCS, in SF-36), based on “minimal improvement” in the validated ACR/EULAR Myositis Response Criteria [37]. The statistical significance level was set to 0.05 (two-tailed) with a statistical power of 80% and an anticipated dropout rate of 10%. To achieve this, a total of 60 participants (30 in each intervention arm) were deemed needed for the study (http://www.sealedenvelope.com/power/continuous-superiority/).

#### Statistical analysis

The statistical plan for the present RCT was developed in conjunction with the published protocol [21] prior to the inclusion of the first patient.

Statistical analysis was conducted to examine the change in the primary outcome variable (QoL assessed as PCS in SF-36) from baseline to post-intervention in IG compared to CG. This analysis was conducted using the “intention-to-treat” principle [38] and in the case of missing values, multiple imputations were performed (MICE—R Studio). To evaluate the difference in pre-to-post training changes between the two groups over time, ANCOVA analysis was used for both primary and secondary outcomes with baseline values of the investigated variable, age, disease duration, and medication (yes/no) as covariates. Post hoc analysis of effect size was calculated for significant outcomes of the ANCOVA analysis [39].

Further, paired t-testing was performed to identify within-group changes in IG and CG, respectively. Statistical significance was set at p ≤ 0.05 (two-tailed testing).

As scheduled in the statistical plan [21], we also performed a “per-protocol” analysis excluding participants lost to follow-up (n = 2).

As an additional analysis (not predefined in the statistical plan) the ANCOVA analysis was performed without adjustments for covariates.

All investigated outcome variables are presented as group means and standard deviation, while between-group differences are presented with 95% confidence intervals unless otherwise stated. Baseline demographics and disease characteristics of the study population were determined and reported descriptively. R Studio (V. 1.2.5001) [40] was used for all statistical procedures.

## Results

### Baseline demographics and clinical characteristics

**Fig. 1:**
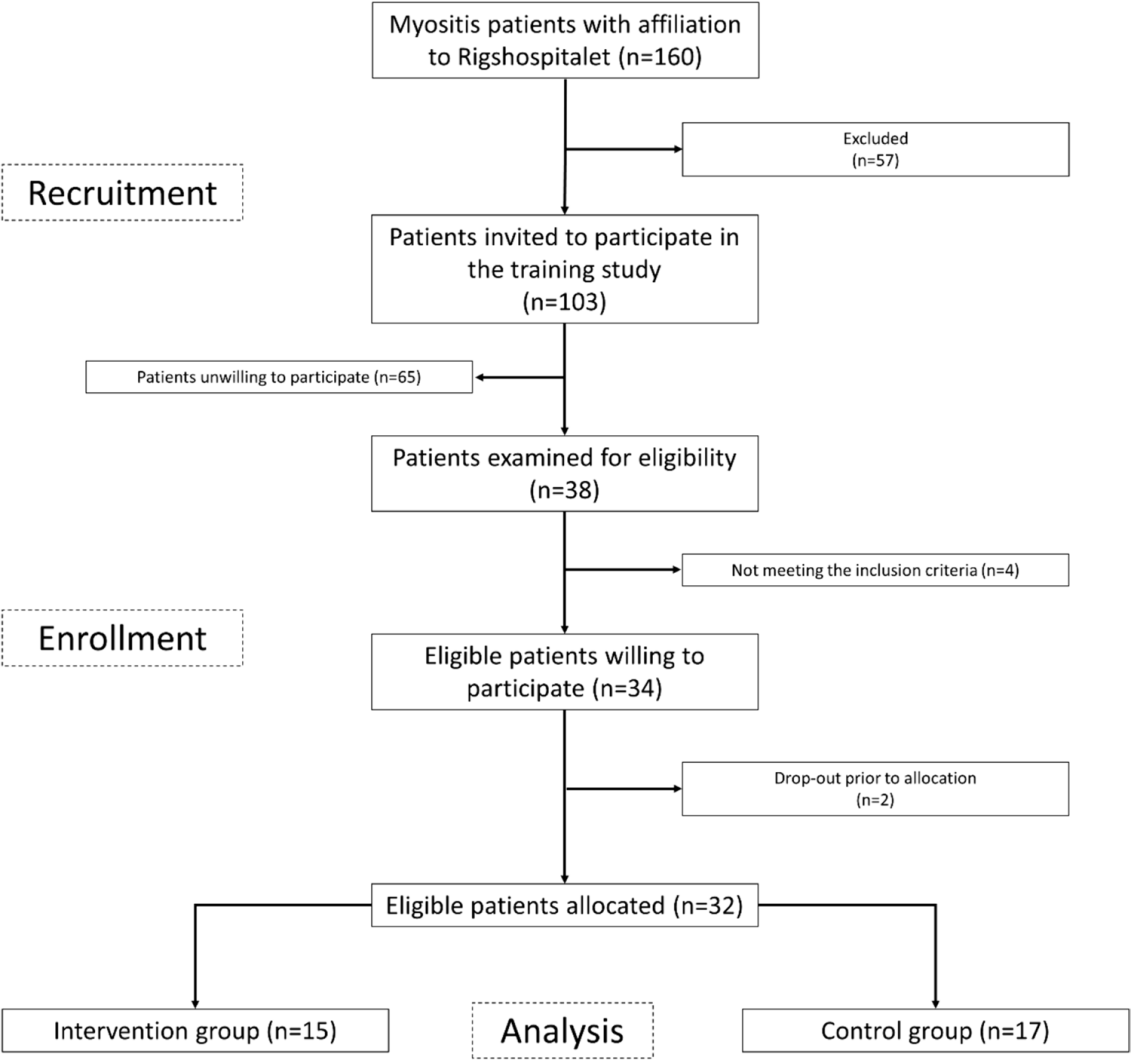
Flowchart of patient recruitment

Following the initial screening, 57 patients were excluded (flowchart depicted in Fig. 1). Consequently, 103 patients were invited to participate in the intervention study. Sixty-five declined to participate, while four patients did not meet the inclusion criteria of ≤ 5 mg of prednisolone administration per day. Two patients dropped out prior to group allocation (1 death and 1 personal circumstances). A total of 32 patients with established and stable myositis were randomly allocated to the intervention group (IG) (n = 15) or the control group (CG) (n = 17). Baseline demographics and clinical characteristics are shown in Table 1.

**Table 1:** Baseline demographics, clinical characteristics, and core set measures of participating patients with myositis

	IG (n = 15)	CG (n = 17)
Female, n (%)	10 (66.7%)	11 (68.7%)
Age, years	44.9 ± 18.9	50.3 ± 14.7
Caucasian, n (%)	15 (100%)	16 (94%)
Disease duration, years	5.8 ± 4.7	4.4 ± 3.5
IIM subset, n (%)		
Dermatomyositis	3 (20.0%)	5 (29.4%)
Amyopathic dermatomyositis	1 (6.7%)	1 (5.9%)
Juvenile dermatomyositis	4 (26.7%)	1 (5.9%)
Antisynthetase syndrome	5 (33.3%)	7 (41.2%)
Immune-mediated necrotizing myopathy	1 (6.7%)	2 (11.8%)
Polymyositis	1 (6.7%)	1 (5.9%)
Myositis-specific autoantibodies, n (%)		
Anti-Jo-1	1 (6.7%)	0 (0%)
Anti-OJ	4 (26.7%)	7 (41.2%)
Anti-SRP	1 (6.7%)	0 (0%)
Anti-HMGCR	0 (0%)	2 (11.8%)
Anti-Mi2	0 (0%)	1 (5.9%)
Anti-NXP2	2 (13.3%)	1 (5.9%)
Anti-MDA-5	1 (6.7%)	1 (5.9%)
Anti-TIF1-γ	1 (6.7%)	2 (11.8%)
Myositis-associated autoantibodies, n (%)		
Anti-Ro	1 (26.7%)	6 (35.3%)
Anti-Pm/Scl-75	2 (13.3%)	1 (5.9%)
Anti-Pm/Scl-100	1 (6.7%)	1 (5.9%)
Extramuscular organ involvement		
Interstitial lung disease, n (%)	8 (53.3%)	10 (58.8%)
Arthritis, n (%)	8 (53.3%)	10 (58.8%)
Raynaud, n (%)	7 (46.7%)	5 (29.4%)
Dysphagia, n (%)	9 (60.0%)	8 (47.1%)
Disease activity measures		
Creatine kinase, mmol/L Reference value: 40–280 mmol/L	277 ± 460	162 ± 172
Health assessment questionnaire, 0–3	0.21 ± 0.29	0.29 ± 0.61
Manuel muscle testing 8, 0–80	76.7 ± 3.9	76.9 ± 2.6
Physician global activity, VAS 0–100 mm	7.9 ± 8.8	6.6 ± 6.5
Patient global activity, VAS 0–100 mm	4.3 ± 5.0	3.2 ± 3.5
Extramuscular global assessment, VAS 0–100 mm	4.0 ± 5.4	3.2 ± 3.5
Disease damage measures		
Physician global damage, VAS 0–100 mm	17.0 ± 13.2	15.0 ± 9.4
Patient global damage, VAS 0–100 mm	19.7 ± 18.9	18.2 ± 14.1
Immunosuppressives, n (%)	11 (73.3%)	15 (82.4%)
Prednisolone	3^a^	2^c^
sDMARD	10	13
bDMARD	3^b^	3^d^

Data are presented as mean ± SD or number & (%)

*IG* group trained with high-intensity resistance training, *CG* control group, *sDMARD* synthetic disease-modifying antirheumatic drugs, *bDMARD* biological disease-modifying antirheumatic drugs

^a^All patients who received prednisolone also received sDMARD. ^b^All patients who received bDMARD also received sDMARD, while two out of three also received prednisolone. ^c^One patient who received prednisolone also received sDMARD, while the other received bDMARD. ^d^One patient received bDMARD only

One patient allocated to IG discontinued the training during the intervention period, due to a lack of energy to engage in training twice a week. One patient in CG was withdrawn from the study, as immunosuppressive treatment had to be discontinued, due to severe side effects caused by the immunosuppressive treatment.

The mean attendance rate in IG was 27.7 ± 3.0 training sessions, corresponding to an adherence of 87 ± 9%. During the training intervention, there were twenty COVID-19 related cancellations of planned training sessions. The COVID-adjusted training adherence was 90%.

### Primary outcome

**Fig. 2:**
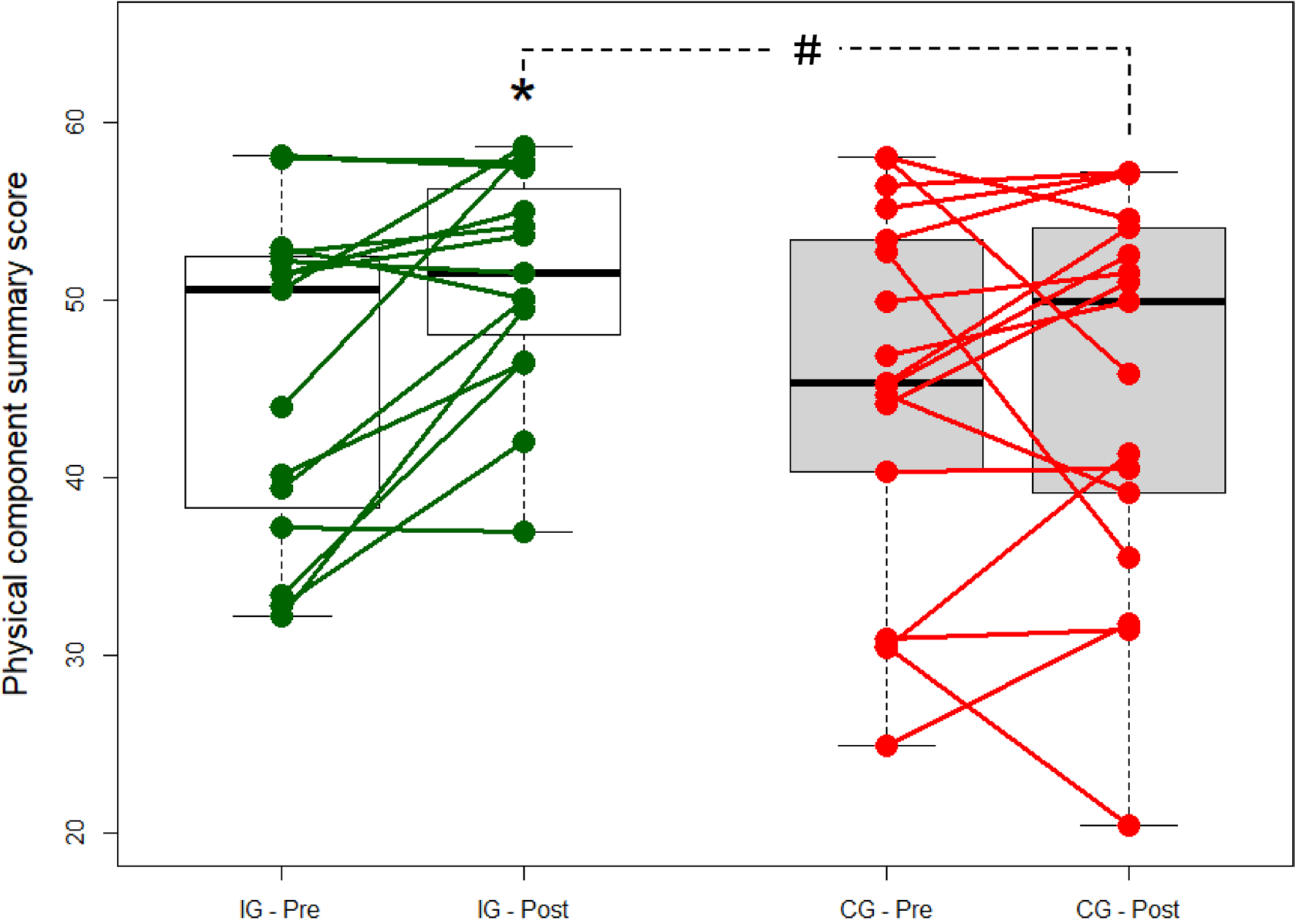
Changes in primary outcome—physical component summary of SF36. *Symbolising a significant with-in group difference (p < 0.05). ^#^Symbolising a significant between-group difference (p < 0.05)

QoL assessed as SF-36 PCS showed a between-group difference in the change over time of 5.33 (0.61; 10.05) (p = 0.03) favouring HRT, with an effect size of *d* = 0.521. Within IG, PCS increased 12.0% following the 16-week intervention period, from 45.8 ± 9.2 to 51.3 ± 6.3 (p = 0.005). In contrast, PCS remained unchanged in CG, 45.1 ± 10.5 vs. 45.4 ± 10.9 (p = 0.90) (Fig. 2).

### Secondary outcomes

#### Quality of life—MCS

SF-36 MCS did not differ between groups over time 0.1 (− 7.5; 7.7) (p = 0.98). MCS remained unaltered in IG: 48.4 ± 14.0 vs. 51.1 ± 8.1 (p = 0.46), as well as in CG: 43.5 ± 13.7 vs. 47.7 ± 15.2 (p = 0.08).

#### Functional capacity, muscle power and strength

FI3 showed a between-group difference in the magnitude of pre-to-post change over time of 11.49 (3.37; 19.60) (p = 0.007) in favour of HRT, with an effect size of *d* = 0.425. In terms of within-group changes, FI3 increased by 29.6% following HRT (p = 0.0004), with no changes in CG (Table 2).

**Table 2:** Changes in functional performance and maximal leg extensor muscle power pre-to-post intervention in the exercising (IG) and non-exercising (CG) groups

	IG Pre	IG Post	Within-group	CG Pre	CG Post	Within-group	Between-group difference	
	Mean (SD)	Mean (SD)	P-value	Mean (SD)	Mean (SD)	P-value	Difference (95% CI)	P-value
FI3 (%)	56.8 (30.5)	73.6 (28.8)	**0.0004**	71.1 (27.9)	75.2 (24.6)	0.10	**11.49** **(3.37; 19.60)**	**0.007**
30-s STS (repetitions)	15.5 (4.4)	16.5 (5.2)	**0.03**	15.0 (4.2)	15.1 (5.2)	0.78	0.80 (-0.50; 2.10)	0.22
TUG (s)	6.2 (1.5)	5.4 (1.3)	** < 0.0001**	5.9 (1.9)	5.7 (1.8)	0.45	-0.38 (-0.98; 0.21)	0.19
2MWT (m)	197.1 (29.5)	206.7 (29.7)	**0.008**	194.9 (35.1)	202.7 (35.1)	**0.02**	0.20 (-8.72; 9.12)	0.96
Balance (s)	29.6 (1.4)	29.8 (0.7)	0.51	29.1 (2.7)	29.7 (1.0)	0.23	-0.13 (-0.59; 0.34)	0.58
LEP (W/kg)	2.2 (0.8)	2.50 (1.1)	**0.04**	2.7 (1.1)	2.7 (1.2)	0.60	0.19 (-2.17; 0.59)	0.35

Data are presented as group means and standard deviation. Between-group difference is presented with 95% confidence intervals in parentheses. Bold font means significant

*FI3* functional index 3, *30-s STS* 30-s sit-to-stand, *TUG* timed up and go, *2MWT* 2-min walk test, *LEP* leg extensor power

HRT demonstrated significant within-group improvements following the intervention period for all functional measures, except for postural balance. Thus, 30-s STS increased 6.5% (p = 0.03), TUG improved 12.9% (p < 0.0001), 2MWT increased 4.9% (p = 0.008) and LEP increased 14.2% (p = 0.04). In CG, 2MWT increased 4.0% (p = 0.02), while all other measures of functional capacity remained unchanged (Table 2).

10-RM muscle strength improved (p < 0.0001) following HRT in all five training exercises (43–95%) (Table 3).

**Table 3:** Changes in 10 RM muscle strength pre-to-post intervention in the exercising group (IG)

	IG session 5	IG session 32	Within-group (p-value)
Bench press (kg)	13.6 (8.8:18.3)	25.5 (18.2:32.8)	** < 0.0001**
Leg press (kg)	55.7 (43.1:68.3)	79.6 (66.4:92.9)	** < 0.0001**
Cable row (kg)	27.1 (19.7:34.5)	41.6 (30.8:52.4)	** < 0.0001**
Knee extension (kg)	14.6 (9.6:19.6)	28.5 (19.5:37.5)	**0.0002**
Bicep curls (kg)	4.1 (3.1:5.0)	6.0 (4.7:7.3)	** < 0.0001**

Data are presented as group means (95% confidence intervals). Bold font means significant

*RM* repetitions maximum

### IMACS disease activity core set measures

MMT8 showed a significant between-group difference in the magnitude of pre-to-post difference, favouring HRT by 1.30 (0.09; 2.51) (p = 0.04), with an effect size of *d* = 0.447. Within the IG, MMT8 increased 2.4% (p = 0.02) following the intervention period with no changes in CG (Table 4).

**Table 4:** Changes in IMACS disease activity core set measures pre-to-post intervention in the exercising (IG) and non-exercising (CG) groups

	IG Pre	IG Post	Within-group	CG Pre	CG Post	Within-group	Between-group difference	
	Mean (SD)	Mean (SD)	p-value	Mean (SD)	Mean (SD)	p-value	Difference (95% CI)	p-value
PhGA (0–100)	4.3 (5.0)	4.6 (6.7)	p = 0.79	3.2 (3.5)	4.0 (5.7)	p = 0.57	− 0.46 (− 4.49; 3.57)	p = 0.82
PtGA (0–100)	7.9 (8.8)	6.7 (10.9)	p = 0.51	6.6 (6.5)	6.6 (9.6)	p = 0.99	− 1.30 (− 7.13; 4.53)	p = 0.65
EMGA (0–100)	4.0 (5.4)	4.0 (7.0)	p = 0.97	3.2 (3.5)	3.6 (5.7)	p = 0.77	− 0.33 (− 4.21; 3.56)	p = 0.86
MMT8 (0–80)	76.7 (3.9)	78.5 (2.0)	**p = 0.02**	76.9 (2.6)	77.2 (2.7)	p = 0.51	**1.30** **(0.09; 2.51)**	**p = 0.04**
HAQ (0–3)	0.208 (0.290)	0.094 (0.172)	p = 0.08	0.294 (0.609)	0.241 (0.347)	p = 0.51	− 0.09 (− 0.20; 0.02)	p = 0.12
CK (mmol/L)	277 (460)	146 (88)	p = 0.27	162 (172)	222 (352)	p = 0.50	− 103 (− 313; 107)	p = 0.33
PhGD (0–100)	17.0 (13.2)	12.3 (9.4)	**p = 0.02**	15.0 (9.4)	13.9 (9.4)	p = 0.49	− 3.34 (− 7.63; 0.95)	p = 0.12
PtGD (0–100)	19.7 (18.9)	10.4 (8.2)	**p = 0.03**	18.2 (14.1)	13.9 (10.8)	p = 0.07	− 4.84 (− 10.19; 0.51)	p = 0.07

Data are presented as means and standard deviation. Between-group difference is presented with 95% confidence intervals in parentheses. Significant values are in bold font

*PhGA* physician global activity, *PtGA* patient global Activity, *EMGA* extramuscular global assessment, *MMT8* manual muscle testing 8, *HAQ* Health assessment questionnaire, *CK* creatine kinase, *PhGD* physician global damage, *PtGD* patient global damage

HAQ tended to improve in IG (p = 0.08) while remaining unaltered in CG (Table 4).

PhGA, PtGA, EMGA, and CK remained constant from pre-to-post intervention in both groups (Table 4).

PhGD and PtGD were found to improve in IG (− 27.7% and − 47.2%, respectively) following the intervention period, with no changes observed in CG (Table 4).

### Adverse events

In IG, a total of 43 adverse events (AEs) were registered, of which 42 were categorised as”non-serious” and “excepted”. A single case was categorised as”non-serious” and “excepted”, which was a case of non-acute fatigue.

Acute low-level physical fatigue was the most prevalent AE, with 30 events recorded of which 14 (47%) were deemed “study-related”. Incidents of situational pain (short-term pain in certain movements) were noted 10 times with 3 (30%) of these categorised as “study-related”. Lastly, muscle soreness was reported twice, categorised as “study-related”.

In CG, one “serious” and “unexpected” AE was recorded due to severe side effects caused by the immunosuppressive treatment.

### Per protocol analysis

No differences were found between our “intention-to-treat” and the “per protocol” analyses.

ANCOVA analysis without correcting for covariates showed no differences in statistical significance compared to the presented data.

## Discussion

In the present study, we examined the efficacy of stand-alone high-intensity resistance training on quality of life, muscle strength and functional capacity in patients with myositis. As the main observations, 16 weeks of high-intensity resistance training produced marked improvements in quality of life compared to non-trained control patients. Importantly also, the HRT intervention led to significant improvements in functional capacity, muscle endurance and clinical measures of muscle strength, respectively. Finally, the HRT intervention protocol did not incite disease flare-ups in the present cohort of myositis patients.

While we did not fully reach our intended 20% change in QoL assessed by the SF-36 physical component summary score, the present HRT intervention did yield noteworthy improvements (+ 12%) in PCS score, which were not observed in the control group. Notably, this improvement was achieved despite relatively high baseline PCS scores (~ 46 ± 9) compared to previously reported (36.5 ± 9.5) in a comparable Danish cohort of patients with myositis [7]. Rider et al. have defined clinical improvement criteria in patients with myositis [2], however, no study so far has quantified the minimal important difference for SF-36 PCS in myositis. In systemic lupus erythematosus, a minimally important difference of 2.1 points in PCS score has been suggested previously [41]. In the present study, PCS increased by 5.5 points (1.6-fold higher than 2.1 points) following 16 weeks of HRT, which suggests an improvement potentially crossing the threshold for clinical significance, thus holding relevance for individual patients with myositis.

Previously, only a single exercise-based case-series study (n = 3) in myositis patients performing concurrent aerobic and resistance exercise training has been able to demonstrate significant improvements in both physical and mental QoL (PCS and MCS scores) [42]. For comparison, other exercise-based studies employing endurance training [15, 43], resistance-related training [44, 45], or combined endurance and resistive training [16, 20] have reported improvements in various SF-36 subdomains related to PCS, without notable changes in MCS. In consensus with these observations, the present high-intensity resistance training protocol was able to positively affect the physical component summary score, while the mental component summary score remained unaffected. To further strengthen this point, our baseline analysis showed multiple correlations between strength, physical function and PCS, while no correlations to MCS [46].

The present increase in clinically assessed muscle strength (MMT8) following 16 weeks of high-intensity resistance training is closely supported by the observed gains in 10RM muscle strength (cf. Table 3). Previous studies examining the physiological effects of physical exercise in patients with myositis also have reported gains in MMT8, interventions ranging from 7 weeks to 6 months [20, 43–45, 47, 48].

In the present study, participants allocated to HRT demonstrated marked improvements in all measures of functional capacity, apart from postural balance. However, only muscle endurance (FI3) was found to improve significantly compared to participants receiving usual care. Increases in muscle endurance previously have been reported in response to multi-exercise endurance and/or resistance-based training in myositis patients [20, 43–45, 47, 48].

Even though a single participant dropped out of the training group due to fatigue, only minor Adverse Events (AEs) were noted post-training sessions, such as acute low-level physical fatigue, situational pain, and muscle soreness, which align with expectations for healthy adults [49]. Further, the present 16-week resistance training protocol did not induce any adverse effects in terms of heightened disease activity or signs of musculoskeletal damage. Similar observations have previously been reported in the exercise literature (including endurance, resistive and combined exercise protocols) [20, 42, 44, 50, 51]. Collectively, the available data thus indicate that physical training including high-intensity resistance training may be considered safe for patients with myositis.

A number of limitations may be mentioned for the present study. Due to restrictions imposed by the COVID-19 pandemic, the study was postponed for 6 months. Despite this delay, the targeted participant enrollment was not achieved, as some invited patients declined participation due to concerns about being infected by COVID-19.

In combination with the high inter-individual variability observed for a majority of the outcome variables, it may have rendered the current RCT statistically underpowered, albeit significant outcomes demonstrated effect sizes ranging from small to medium (*d* = 0.431–0.602).

The recruitment of myositis patients may have been unintentionally skewed in the present study. The included patients had established myositis with high baseline performance in HAQ and as well as TUG and 30-s STS, altogether reflecting high levels of functional capacity and physical activity prior to entering the study. Thus, patients with lower functional capacity and muscle strength expected to benefit the most from a structured high-intensity resistance training program were generally not included in the present study. Despite these potential limitations, we observed significantly better clinical responses in our primary outcome, MMT8 and FI3 in the exercising group.

Unlike all other outcome assessors, the investigator overseeing the evaluation of FI3 was not blinded to group allocation (training supervision), which makes it impossible to rule out unconscious assessor bias.

As a common trait in physical intervention studies, improvements were also observed in the control group, possibly stemming from behavioural modifications in physical activity [52]. This phenomenon is likely to attenuate some of the between-group disparities observed in all functional and muscle power-related outcomes.

In the design of this RCT, a follow-up period was not included initially, however, an evaluation of the long-term effects of high-intensity resistance training has subsequently been conducted (not published).

Several strengths warrant acknowledgement in this study. The present data suggest that high-intensity resistance exercise training is both feasible and well-tolerated in patients with myositis performing a regiment of two training sessions per week using adjustable weight training machines. Extending this notion, IG training compliance was high (87%).

The present trial was conducted as a single-centre study with all tests and analyses performed by the same assessors (i.e., same physician, same physiotherapist) pre-and post-intervention. This approach significantly mitigated the potential for inter-rater bias. Likewise, all training was supervised by a single trained exercise physiologist, which minimised possible inconsistencies in the training procedures.

All participants were included at the same time point, which inherently eliminated any between-group differences related to continuous seasonal changes throughout the time course of the study, hence reducing the amount of inter-group bias potentially arising from this factor.

## Conclusion

The present study is the first RCT to investigate the effect of high-intensity resistance training in myositis patients. As our main finding, physical quality of life was improved following 16 weeks of high-intensity resistance training compared to non-training control patients receiving usual care. The observed improvement in QoL was accompanied by parallel gains in muscle endurance and clinical measures of muscle strength, respectively. Importantly, disease activity remained unaffected following the intervention period, indicating that high-intensity resistance training is feasible and well-tolerated while eliciting positive clinical effects in patients with stable myositis.

## Data Availability

Researchers interested in collaborating using the data from the study should contact the corresponding author and senior (last) author.

## Declarations

### Congress abstracts

K. Jensen, P. Aagaard, C. Suetta, J. Nielsen, R. Bech, H. Schrøder, J. Christensen, L. Diederichsen. High-intensity strength training improves quality of life, muscle endurance and strength in patients with myositis. 39th Scandinavian Conference of Rheumatology (SCR), OP14. ORAL PRESENTATIONS. (2023). Scandinavian Journal of Rheumatology 52 (sup131): 9–59. 10.1080/03009742.2023.2233370.

Jensen K, Aagaard P, Suetta C, Nielsen J, Bech R, Schrøder H, Simonsen C, Christensen J, Diederichsen L. High-intensity Resistance Training Improves Quality of Life, Muscle Endurance and Strength in Patients with Myositis [abstract]. Arthritis Rheumatol. 2023; 75 (suppl 9). https://acrabstracts.org/abstract/high-intensity-resistance-training-improves-quality-of-life-muscle-endurance-and-strength-in-patients-with-myositis/. Accessed July 29, 2024.
